# Benefits of Aldosterone Receptor Antagonism in Chronic Kidney Disease (BARACK D) trial–a multi-centre, prospective, randomised, open, blinded end-point, 36-month study of 2,616 patients within primary care with stage 3b chronic kidney disease to compare the efficacy of spironolactone 25 mg once daily in addition to routine care on mortality and cardiovascular outcomes versus routine care alone: study protocol for a randomized controlled trial

**DOI:** 10.1186/1745-6215-15-160

**Published:** 2014-05-08

**Authors:** Nathan R Hill, Daniel Lasserson, Ben Thompson, Rafael Perera-Salazar, Jane Wolstenholme, Peter Bower, Thomas Blakeman, David Fitzmaurice, Paul Little, Gene Feder, Nadeem Qureshi, Maarten Taal, Jonathan Townend, Charles Ferro, Richard McManus, FD Richard Hobbs

**Affiliations:** 1 Nuffield Department of Primary Care Health Sciences, University of Oxford, Radcliffe Observatory Quarter, Woodstock Road, Oxford OX2 6GG, UK; 2 NIHR Oxford Biomedical Research Centre, Oxford University Hospitals NHS Trust, John Radcliffe Hospital, Headley Way, Oxford OX3 9DU, UK; 3 Department of Public Health, University of Oxford, Old Road Campus, Headington, Oxford OX3 7LF, UK; 4 Centre for Primary Care, Institute of Population Health, University of Manchester, Williamson Building, Oxford Road, Manchester M13 9PL, UK; 5 Primary Care Clinical Sciences, School of Health and Population Sciences, College of Medical and Dental Sciences, University of Birmingham, Edgbaston, Birmingham B15 2TT, UK; 6 Primary Medical Care, University of Southampton, Aldermoor Health Centre, Aldermoor Close, Southampton SO16 5ST, UK; 7 School of Social and Community Medicine, University of Bristol, Office Room 1.01c, Canynge Hall, 39 Whatley Road, Bristol BS8 2PS, UK; 8 School of Medicine, Room 1307 Tower Building, University Park, Nottingham NG7 2RD, UK; 9 Department of Renal Medicine, Royal Derby Hospital, Uttoxeter Road, Derby, Derbyshire DE22 3NE, UK; 10 Cardio-Renal Research Group, Departments of Cardiology and Nephrology, Queen Elizabeth Hospital Birmingham and University of Birmingham, Edgbaston, Birmingham B15 2TH, UK

**Keywords:** Cardiovascular disease, Chronic kidney disease, CKD mortality, CKD aldosterone receptor antagonist

## Abstract

**Background:**

Chronic kidney disease (CKD) is common and increasing in prevalence. Cardiovascular disease (CVD) is a major cause of morbidity and death in CKD, though of a different phenotype to the general CVD population. Few therapies have proved effective in modifying the increased CVD risk or rate of renal decline in CKD. There are accumulating data that aldosterone receptor antagonists (ARA) may offer cardio-protection and delay renal impairment in patients with the CV phenotype in CKD. The use of ARA in CKD has therefore been increasingly advocated. However, no large study of ARA with renal or CVD outcomes is underway.

**Methods:**

The study is a prospective randomised open blinded endpoint (PROBE) trial set in primary care where patients will mainly be identified by their GPs or from existing CKD lists. They will be invited if they have been formally diagnosed with CKD stage 3b or there is evidence of stage 3b CKD from blood results (eGFR 30–44 mL/min/1.73 m^2^) and fulfil the other inclusion/exclusion criteria. Patients will be randomised to either spironolactone 25 mg once daily in addition to routine care or routine care alone and followed-up for 36 months.

**Discussion:**

BARACK D is a PROBE trial to determine the effect of ARA on mortality and cardiovascular outcomes (onset or progression of CVD) in patients with stage 3b CKD.

**Trial registration:**

EudraCT: 2012-002672-13

ISRTN: ISRCTN44522369

## Background

Chronic kidney disease (CKD) is increasingly common, affecting around 6% of the UK population [1,2], associated with an age-related decline in renal function that is accelerated in hypertension, diabetes mellitus, obesity, and primary renal disorders [3]. While this high (and rising [4]) prevalence is in part due to the ageing population, it is also a result of increases in hypertension and diabetes mellitus. CKD is defined and categorised in to five stages using estimated glomerular filtration (eGFR) as well as evidence of renal damage (imaging or proteinuria) in the early stages [5]. The largest group, with over 90% of cases, is estimated to be CKD stage 3 with 84% stage 3a (eGFR of 45 to 59 mL/min/1.73 m^2^[2]) and 16% stage 3b (eGFR of 30 to 44 mL/min/1.73 m^2^[6]). Population studies have used the four-variable modification of diet in renal disease (MDRD) formula to determine eGFR [7]. In patients aged 65 or over, up to 35% have an eGFR of less than 60 mL/min/1.73 m^2^[8]. CKD prevalence appears to be increasing. Data from the American National Health and Nutrition Examination Survey determined that in the period 1999 to 2004 the overall prevalence of CKD stages 1 to 4 increased significantly when compared to the survey period 1988 to 1994 (13.1% versus 10.0%) [4,9,10].

CKD is a major cause of increased mortality and morbidity through increased vascular events and progression to end stage renal failure (ESRF) [11]. These increased events result in CKD incurring a high cost to healthcare systems, with the dialysis required in ESRF benchmarked at the maximum acceptable cost effectiveness threshold for an intervention by most healthcare systems. However, the most important component of CKD in terms of mortality and morbidity is cardiovascular disease (CVD) [12]. There is a graded inverse relationship between cardiovascular risk and eGFR, independent of age, sex, and other risk factors [4,13-15]. While the CVD risk in ESRF is high, the healthcare burden resides in early stages of disease as it is more prevalent, affecting around 35% of those aged over 70 years [16].

Although the risk of coronary artery disease is increased in CKD, the pattern of CVD is atypical, with a much greater incidence of heart failure and sudden cardiac death than in the general CVD population [17-19]. The main pathological features in CKD that appear to determine this particular cardiovascular risk phenotype are: i) left ventricular (LV) hypertrophy and fibrosis accompanied by both systolic and diastolic dysfunction, and ii) arterial wall thickening, stiffening, and calcification (atherosclerosis). Therefore, although patients with CKD also suffer typical patterns of CVD (coronary and peripheral artery atherosclerosis), the excess rates of cardiovascular events in CKD appear to relate more to vascular wall and ventricular changes than to atherosclerosis.

Given this particular vascular pathophysiology, it is unsurprising that conventional cardiovascular risk factors are less predictive of outcomes in CKD than in the general population [20], and less predictive than eGFR and protein excretion [13,15,21], even after controlling for variables such as blood pressure [22]. The 2014 draft update National Institute for Health and Care Excellence (NICE) guidelines for CKD [23] are unable to agree a specific risk assessment tool for patients with CKD stage 3 or above. Furthermore, interventions to reduce the increased cardiovascular risk in CKD have proved disappointing, with only limited evidence for traditional therapies in terms of cardiovascular outcomes.

For example, the SHARP (Study of Heart and Renal Protection) trial [24] aimed to assess the safety and efficacy of reducing LDL cholesterol in more than 9,000 patients with CKD with a low dose of a statin (simvastatin 20 mg daily) and ezetimibe. The trial showed that lowering of LDL cholesterol safely reduced the risk of major atherosclerotic events in patients with CKD. However, the reduction in non-fatal myocardial infarction or coronary death was not significant.

Alternative treatment options to provide protection from vascular events or delay progression of CKD are therefore urgently needed, especially given the increasing burden of the disease.

### Rationale

CKD is common and increasing in prevalence. CVD is a major cause of morbidity and death in CKD, though of a different phenotype to the general CVD population. Currently, few therapies have proved effective in modifying the increased CVD risk or the rate of renal decline in CKD. There are accumulating data that aldosterone receptor blockers (ARA) may offer cardio-protection and delay renal impairment in patients with CKD.

There are recent data that indicate beneficial effects of ARA therapy on surrogate markers for CVD risk in patients with CKD, i.e., not just in those with established advanced CVD such as heart failure. This is important because there are presently limited therapeutic options to reduce overall cardiovascular risk in CKD, with modest effects of LDL reduction shown in the recent SHARP study [24] and sub-studies of large angiotensin-converting-enzyme (ACE) inhibitor and statin trials only suggesting limited cardiovascular benefits in patients with early stage CKD [25,26].

The Birmingham CRIB-2 study, in which two of the present authors were involved (CF & JT), recently showed that the ARA spironolactone provided significant beneficial effects on validated intermediate cardiovascular endpoints of prognostic value, including LV mass and arterial stiffness [27]. In a placebo-controlled, double-blind trial 112 patients with stage 2 and 3 CKD with good blood pressure (BP) control on established treatment with ACE inhibitors or Angiotensin II Receptor Blockers (ARB) were treated in an active run-in phase with spironolactone 25 mg once daily and then randomised to continue spironolactone or to receive a matching placebo. Compared with placebo, the use of spironolactone resulted in highly significant reductions in LV mass and arterial stiffness (pulse wave velocity, augmentation index, and aortic distensibility), and improved myocardial diastolic function and collagen turnover [27]. These clinical findings were attributed to a reduction in arterial and myocardial inflammation and fibrosis but may also be a function of the considerable human and animal evidence base that aldosterone receptor antagonism improves endothelial-dependent vasodilatation and vascular nitric oxide bioactivity [28]. Further, recent data have shown that ARA therapy in non-diabetic CKD haemodialysis patients prevented progression of carotid intima-media thickness [29]. These recent clinical data on the effect of ARA on intermediate vascular outcomes have resulted in calls for definitive trials [30,31].

ARA therapy might therefore be an effective candidate for improved cardiovascular outcomes, through the prevention of aldosterone-mediated vascular endothelial dysfunction as well as widespread cardiovascular inflammation, fibrosis, and hypertrophy. Since spironolactone is well recognised as an effective anti-hypertensive agent for patients with hypertension, even when this is resistant to other drugs [32], the intensive phenotyping of blood pressure, LV function, and arterial stiffness in the Benefits of Aldosterone Receptor Antagonism in Chronic Kidney Disease (BARACK D) trial will enable modelling of the extent to which any positive results may be explained by any BP differences between study arms. The 25 mg dose of spironolactone used in BARACK D, and most clinical trials in which it has been involved, is similar to that used in hypertension and heart failure cases which are states characterised by excess cardiovascular risk and with a high probability of co-morbid CKD.

### Choice of comparators

Blockade of the renin-angiotensin-aldosterone system by ACE inhibitors and ARBs has shown mortality benefit in patients with chronic heart failure and in those with, or at high risk of, coronary artery disease [25,33,34]. The benefits are attributed to prevention of the multiple adverse effects (AEs) of angiotensin II. ACE and ARB’s appear superior to other BP lowering drugs in slowing the progression of CKD, though the effect may be marginal [33]. These agents are therefore widely recommended in international guidelines [35-37] as ‘reno-protection’ for CKD patients, especially those with proteinuria or diabetes mellitus.

Aldosterone may also be an important mediator of cardiac and vascular damage in many disease states. Mineralocorticoid receptors are present in many tissues, including the brain, heart, and blood vessels, as well as the kidney, and there is aldosterone production within these tissues [38]. These receptors may also be activated by circulating glucocorticoids in the presence of oxidative stress [39]. Local mineralocorticoid receptor activation by aldosterone leads to numerous pathological effects on the cardiovascular system including endothelial injury, inflammation, oxidative stress, and fibrosis in the heart and vasculature, as well as the development of hypertension and autonomic dysfunction [38,40,41].

### Rationale for ARA intervention to reduce cardiovascular events

In humans, primary aldosteronism is associated with a greater LV mass and higher risk of adverse cardiovascular events than control hypertensive populations and, in patients after myocardial infarction, plasma aldosterone concentration within the normal range predicts an adverse prognosis [42-44]. A recent study of subjects undergoing coronary angiography confirmed an independent association of plasma aldosterone levels with total and cardiovascular mortality [45].

Renal specialists have avoided the use of ARA drugs because of a perceived risk of azotaemia and hyperkalaemia, though similar restrictions were applied to ACE inhibitors until outcome data were reported. There are, however, accumulating data on combined treatment with ACE and ARA to improve renal function in patients with CKD [46]. From a safety perspective, even oligoanuric haemodialysis patients can tolerate spironolactone in low doses [47].

### Objectives

#### Primary objective

To determine the effect of ARA on mortality and cardiovascular outcomes (onset or progression of CVD) in patients with stage 3b CKD.

#### Secondary objectives

To determine the effect of ARA in patients on measures of: cardiovascular haemodynamics; LV function; decline in renal function; treatment costs and benefits; and incidence of transient ischaemic attack; and to determine the safety of ARA in patients with stage 3b CKD.

#### Intensively phenotyped group

To determine the effect of ARA in patients on measures of cardiovascular haemodynamics.

### Trial design

A prospective, randomised, open, blinded endpoint (PROBE) trial: eligible patients, from 120 practices recruited by 6 National Institute for Health Research School for Primary Care Research departments in addition to a specialist renal trial recruitment group, with previously recorded blood test results suggesting CKD stage 3b will be invited to take part in the study and randomised to either spironolactone 25 mg once daily in addition to routine care or routine care alone. BP in both groups will be titrated (monitored and adjusted accordingly) by the clinicians against NICE guideline standards and checks of electrolytes undertaken.

A subgroup of participants will form the intensively phenotyped group in whom 24-h BP and arterial stiffness will be monitored in detail to enable modelling of the extent to which positive results may be explained by any BP differences between study arms. The secondary endpoints assessed in the intensively phenotyped group will determine the effect of ARA on 24-h BP and pulse wave velocity.

## Methods

The study is set in primary care where patients will primarily be identified opportunistically by their primary care clinician or systematically from existing CKD lists. They will be invited if they have been formally diagnosed with CKD stage 3b or there is evidence of stage 3b CKD from blood results (eGFR 30–44 mL/min/1.73 m^2^[2]) and fulfil the other inclusion/exclusion criteria.

Potentially eligible patients will be invited to attend a baseline clinic at their own practice where the trial will be explained. Informed consent will be obtained and baseline assessments performed.

A subset of patients will form the intensively phenotyped group who will undergo additional trial procedures as described below and in the procedure schedule shown in Table 1. The intensive phenotyping of 24-h BP and arterial stiffness in BARACK D will enable modelling of the extent to which any positive results may be explained by any BP differences between study arms.

**Table 1:** BARACK D patient visit schedule

	**Treatment and follow-up**																
**Week**	**B**	**0**	**1**	**2**	**4**	**12**	**26**	**39**	**52**	**65**	**78**	**91**	**104**	**117**	**130**	**143**	**156**
**Visit**	**V**		**V1**	**V2**	**V3**	**V4**	**V5**	**V6**	**V7**	**V8**	**V9**	**V10**	**V11**	**V12**	**V13**	**V14**	**V15**
Valid informed consent	**x**	**Randomisation *in absentia *** **and prescription produced once blood results received**															
Full demographic details	**x**																
Medical history	**x**																**x**
Clinical history	**x**																
Concomitant medications	**x**					**x**	**x**		**x**		**x**		**x**		**x**		**x**
Weight, height, waist/hip	**x**																**x**
Physical examination	**x**																
Office blood pressure measurement	**x**		**x**	**x**	**x**	**x**	**x**	**x**	**x**	**x**	**x**	**x**	**x**	**x**	**x**	**x**	**x**
Home blood pressure measurement						**x**			**x**		**x**		**x**		**x**		**x**
KDQOL-SF questionnaire*	**x**						**x**		**x**				**x**				**x**
QoL EQ-5D-5 L questionnaire*	**x**						**x**		**x**				**x**				**x**
ICECAP-A questionnaire*	**x**						**x**		**x**				**x**				**x**
QoL VAS*	**x**						**x**		**x**				**x**				**x**
Diary card (medication monitoring)	**x**					**x**	**x**		**x**		**x**		**x**		**x**		**x**
Diary card (Health Economics)	**x**					**x**	**x**	**x**	**x**	**x**	**x**	**x**	**x**	**x**	**x**	**x**	**x**
Adverse event monitoring	**x**		**x**	**x**	**x**	**x**	**x**	**x**	**x**	**x**	**x**	**x**	**x**	**x**	**x**	**x**	**x**
Urine albumin/creatinine ratio	**x**																**x**
12 lead ECG	**x**																**x**
**Blood tests for:**																	
Full blood count	**x**																**x**
Renal profile	**x**		**x**	**x**	**x**	**x**	**x**	**x**	**x**	**x**	**x**	**x**	**x**	**x**	**x**	**x**	**x**
Liver function test and bone profile	**x**						**x**		**x**				**x**				**x**
Lipids	**x**						**x**		**x**				**x**				**x**
HbA1c	**x**						**x**		**x**				**x**				**x**
Fasting blood sugar	**x**						**x**		**x**				**x**				**x**
B-type natriuretic peptide	**x**						**x**		**x**				**x**				**x**
Future analysis (where applicable)	**x**								**x**				**x**				**x**
**Intensively phenotyped group only**																	
Pulse wave velocity	**x**						**x**		**x**				**x**				**x**
24-h ambulatory blood pressure estimation	**x**						**x**		**x**				**x**				**x**

*Kidney Disease Quality of Life-Short Form (KDQOL-SF), Quality of Life EuroQol, 5 Dimensions, 5 Levels (QoL EQ-5D-5 L) ICEpop CAPability measure for Adults (ICECAP-A), Quality of Life Visual Analogue Scale (QoL VAS).

### Eligibility criteria

#### Inclusion criteria

Participants must fulfil all of the following:

• Participant is willing and able to give informed consent for participation in the study.

• Male or female, aged 18 years or above.

• Evidence of stage 3b CKD using the MDRD equation on at least two occasions, whether or not on the practice chronic disease register.

• Able (in the recruiting clinician’s opinion) and willing to comply with all study requirements.

• Willing to allow his or her General Practitioner and consultant, if appropriate, to be notified of participation in the study.

• Willing to provide contact details to the research team (encompassing recruitment centre and practice staff), for use at any time should the need arise, on trial-related matters.

• If the participant is a female of child-bearing potential, they are willing to ensure effective contraception during the trial period.

#### Exclusion criteria

The participant may not enter the study if ANY of the following apply:

• Female participants who are pregnant, lactating, or planning pregnancy during the course of the study.

• Type 1 diabetes mellitus.

• Terminal disease or felt otherwise unsuitable by their research clinician.

• Clinical diagnosis of chronic heart failure or known LV systolic dysfunction with ejection fraction ≤ 40%.

• Recent myocardial infarction (within 6 months).

• Alcohol or drug abuse.

• Suspected or known current hazardous or harmful drinking, as defined by an alcohol intake of greater than 42 units every week.

• Suspected or known current substance misuse.

• Documented previous hyperkalaemia not thought to be spurious, or intolerance of spironolactone.

• Serum potassium at baseline over 5 mmol/L.

• Documented Addisonian crisis and/or on fludrocortisone.

• Documented symptomatic hypotension or baseline systolic BP under 100 mmHg.

• Recent acute kidney injury or admission for renal failure.

• Albumin/creatinine ratio (ACR) >70 mg/mmol.

• Prescription of medications with known harmful interactions with spironolactone as documented in the British National Formulary including tacrolimus, lithium, and cyclosporine.

• Any other significant disease or disorder which, in the opinion of the recruiting clinician, may either put the participants at risk because of participation in the study, or may influence the result of the study, or the participant’s ability to participate in the study.

### Interventions

Spironolactone has been selected as the trial ARA, to be used in the “Standard Care + Spironolactone” arm, since it has a large evidence base for effective treatment in hypertension and heart failure. There are considerable data from these trials on the drug’s renal safety in high-risk cardiovascular populations. Spironolactone is also the most cost effective ARA available as a generic prescription. Clinical trial labelling will therefore not be required in accordance with Article 14 of the EU clinical trial directive.

Spironolactone 25 mg will be prescribed as a standard NHS FP10 prescription by the study recruiting research clinician using the recruitment sites’ local pharmacies, processes, and systems, also removing the requirement for trial-specific drug accountability mechanisms to be in place. As such, there will be no trial-specific study treatment requirements. The trial treatment regime will be 25 mg spironolactone once daily for the duration of the trial.

### Modifications

Safety monitoring will include the following discontinuation rules:

#### Hyperkalaemia

In RALES, incidence of serious hyperkalaemia was 2% although patients with a creatinine of >221 μmol/L were excluded [22]. In EPHESUS, Eplerenone caused a K^+^ >5.5 mmol/L in 10% of patients with an eGFR of <70 mL/min [38]. In CRIB-2 [48], during the open label run, only one patient was withdrawn due to hyperkalaemia (K^+^ >6.5) – with results later shown to be spurious – and six had a K^+^ of >5.5 mmol/L requiring dose reduction to alternate days. During the double-blind phase only two patients on ARA and two on placebo had a K^+^ of >5.5 mmol/L. For BARACK D, serum K^+^ and creatinine will be checked at all visits. Patients will stop trial medication if systemically unwell due to intercurrent infection, diarrhoea, or need for surgical intervention for any reason. The study drug will be re-started one week after the recruiting research clinician is satisfied recovery has taken place; serum K^+^ and creatinine will be rechecked at weeks 1 and 2 following resumption. The protocol below will be followed in the event of hyperkalaemia:

• Serum potassium below 5.4 mmol/L, no action;

• Between 5.5–5.9, reduce dose to 25 mg alternate days;

• 6.0–6.4 stop study drug and restart after 7 days on alternate days and if remains over 6.0 withdraw patient from trial treatment;

• >6.5 appropriate management and withdraw patient from trial treatment.

#### Deterioration of renal disease

If there is a deterioration of 20% in eGFR between successive visits then trial treatment will be withdrawn and specialist care referral made. Patients will also be withdrawn if there is a reduction in eGFR of 25% from their baseline eGFR, or an increase in creatinine of 30% over the baseline value.

#### Hypotension

If there is >20 mmHg systolic postural drop in BP with symptoms during the trial and/or the systolic BP drops to below 100 mmHg, then the trial medication will be discontinued.

If withdrawn from the trial, the reason for withdrawal will be recorded on the trial withdrawal form and if due to an AE, the research team will arrange for follow-up visits or telephone calls until the AE has resolved or stabilised.

### Adherence

Study treatment compliance will be self-monitored throughout the trial using a medication monitoring diary card. For participants assigned to the spironolactone treatment arm if compliance cannot be verified through patient report, prescription uptake will also be verified by the patient’s research clinician through database searches of prescription collection.

### Concomitant care

If participants on the spironolactone arm develop medical conditions which require treatment with medications known to have harmful interactions with spironolactone as listed in the British National Formulary, then their prescription will be halted [49] but follow-up will continue.

Throughout the trial the participant remains the responsibility of their GP practice and therefore under normal care.

### Outcomes

#### Primary endpoint

Time from randomisation until the first occurring death, first onset, or hospitalisation for heart disease (coronary heart disease, arrhythmia, new onset/first recorded atrial fibrillation, sudden death, failed sudden death), stroke, or heart failure. Primary endpoints will be adjudicated by an independent Endpoint Committee blinded to the treatment arm.

#### Secondary endpoints

• Change in BP annually and at final visit

• Rates of hypotension (<100 mmHg systolic or >20 mmHg systolic drop on standing)

• Changes in B-type natriuretic peptide (BNP)

• Change in urine ACR

• Changes in eGFR

• Change in health-related quality of life on EQ-5D-5 L, ICECAP-A, and QoL-VAS

• Incremental cost effectiveness analysis

• Transient ischaemic attack – as defined by the American Heart Association (2009) [50]

• Rates of AEs

• Rates of hyperkalaemia

#### Intensively phenotyped group

• Mean change in ambulatory BP from randomisation to final visit (measured in mmHg)

• Change in carotid-femoral pulse wave velocity from baseline to final visit

### Participant timeline

Following consent, all patients will have the following information taken and investigations performed at the initial visit: patient demographics, physical examination (height, weight, waist circumference), office BP measurement using a British Hypertension Society validated automated device after 5 minutes rest, venepuncture for routine haematology, and biochemistry including renal function (including eGFR calculated using MDRD and CKD-EPI formulae, hepatic and bone profiles, full blood count, fasting blood sugar, HbA1c, lipids, and BNP), urinalysis using ACR, 12 lead electrocardiograph, and quality of life questionnaires (EQ-5D-5 L, KDQOL-SF, ICECAP-A, and QoL VAS questionnaires). In addition to a diary card to monitor side effects of trial medication, pregnancy tests will be performed on women of childbearing potential, if deemed necessary, at the discretion of the research clinician.

In the intensively phenotyped group only:

• 24-h ambulatory BP estimation

• Pulse wave velocity measured with cardiovascular software, using a validated applanation tonometry device [51]

Following the baseline visit, as with all laboratory analyses returned to the recruitment site under routine care, blood results will be reviewed promptly to ensure safety and eligibility.

Once eligibility is confirmed, the research clinician will randomise the patient (by accessing the Primary Care Clinical Trial Unit’s (PC-CTU) in-house online randomisation system “Sortition” to obtain the randomisation code), produce the necessary prescription if applicable, issue the patient where necessary, and book an appointment for the patient to return for the next visit after taking spironolactone for 7 days or 7 days following randomisation where assigned to the routine care arm.

### Subsequent assessments

Subsequent assessment will continue for both treatment arms for a further 36 months with follow-up visits at weeks 1, 2, 4, 12, 26, and then every 13 weeks to 156 weeks. Windows either side of the visits will be 2 days for V1 and V2, 4 days at V3 and V4, 7 days for V5, and 2 weeks thereafter (all calculated from date of randomisation). Patients will also be flagged with the Office for National Statistics for long-term follow-up of mortality, with initial assessment at 5 years. Measurements at each follow-up visit will vary according to the schedule in Table 1.

Patients will also be supplied with a validated home BP monitoring machine, along with an additional diary card and an instruction sheet, for 1 week every 6 months, to document their self-assessed BPs. They will take 2 readings twice daily, i.e., 2 each morning and 2 each evening over the week. The readings for the first 2 days will be discarded and the mean of the remaining readings taken as the home BP level.

Clinicians will be strongly encouraged to manage BP according to NICE CKD guidelines [37].

### Definition of end of trial

The end of trial will be defined as the date of the last visit for the last participant for the initial 3 year follow-up period. The trial will have an independent Trial Steering Committee (TSC) and Data Monitoring and Ethics Committee (DMEC) who will assess the study feasibility as the trial progresses and will have ‘stop rule’ authority to advise early termination of the trial in the event of safety concerns or futility either through poor recruitment, lack of events, or lack of any treatment effect. These ‘stop rules’ will be defined fully by the DMEC. A formal futility and feasibility analysis will be performed at 12 months by the DMEC to assess recruitment and retention which will determine whether criteria for the trial to proceed have been met.

### Discontinuation/withdrawal of participants from study treatment

Each participant has the right to withdraw from the study at any time in line with the following criteria:

1. Withdrawal from treatment (follow-up continued)

2. Complete withdrawal from trial excluding notes review (without participant involvement)

3. Complete withdrawal

4. In addition, the recruiting research clinician may discontinue a participant from the study treatment at any time if it is considered necessary for any reason. In all cases, where possible, follow-up and inclusion in the intention-to-treat analysis, will continue.

### Sample size

A UK representative spread of practices will be achieved by stratifying practice postcode location into quartiles of Townsend Deprivation Score and selecting practices that agree to take part sequentially until each deprivation quartile practice target is reached. The estimate for the CVD event rate (defined by hospitalisation for coronary heart disease, heart failure, ischemic stroke, and peripheral arterial disease) and total mortality rate in patients with CKD stage 3b being 11.29 and 4.76 per 100 person years, respectively, gives a combined event rate of 16.05 per 100 person years [12]. To detect a 20% relative risk reduction in death or cardiovascular events within 3 years in the intervention group as compared with the control group (i.e., hazard ratio = 0.8) with a two-sided significance of 0.05, 1,308 participants per arm are required seeking 90% power and assuming 10% drop out rate per year.

### Recruitment

Potential subjects will be identified by searching routine electronic clinical records for patients with biochemical evidence of CKD stage 3b (eGFR 30–44 mL/min/1.73 m^2^[2]). The recruitment site will then send out an invitation letter inviting the patients to attend a baseline assessment and eligibility visit. A reply slip, pre-paid envelope, and alternative contact details (e.g., e-mail address and phone number) will be provided for expressions of interest.

For the average GP practice, 180 patients are likely to meet stage 3b CKD criteria. Assuming that around 80% of these patients are eligible and at least 50% of these are willing to take part (based on our experience recruiting to heart failure studies which have a similar age distribution as patients with CKD), then 72 patients may be recruited per practice, requiring 37 practices in total, but increased to 60 to allow for poor recruiting practices, or 15 practices per Townsend quartile of deprivation. To improve the representativeness of the trial population, the number of practices per recruiting centre will be increased to 20 with the intention of reducing these numbers by 50% and giving 30 practices per Townsend quartile of deprivation.

### Sequence generation

Block randomisation with randomly varying block size will be performed in line with PC-CTU standard operating procedures (SOPs) and will be via the internet.

### Concealment mechanism

Randomisation will be performed using Sortition, PC-CTU’s in-house online randomisation system. It supports multiple studies and sites, a range of randomisation algorithms (simple, block, stratified and minimised), unbalanced allocation ratios, blind or open trials, email notifications, and site package statistics (for blind trials). It is secure, provides full audit logs, and has been validated at algorithm and interface levels.

### Implementation

All patients who give consent for participation and who fulfil the inclusion criteria will be randomised. The staff member responsible for recruitment will request randomisation from the PC-CTU via a web interface. Patients will be randomised to treatment with spironolactone 25 mg once daily prescribed on top of routine care or to continue with routine care alone.

### Blinding

BARACK D is a PROBE trial where neither the patients nor research clinicians are blinded to the trial treatment. However, an independent Endpoint Committee, blinded to the treatment arm, will assess the primary endpoints.

### Emergency unblinding

Not applicable as recruitment site remains unblinded.

### Data collection

Source documents will include:

• Primary care electronic and paper records/outputs

• Reports from laboratory investigations

• Hospital correspondence

• Records of 24-h ambulatory and home BP measurements

• Patient questionnaires

• Patient diary cards

• The case report form (CRF) itself where there is no other written or electronic record of data

Clinical trial data is collected by the PC-CTU both electronically and in paper format, with a paper back-up for the data captured electronically.

All documents will be stored safely in confidential conditions according to PC-CTU policies and SOPs. On all study-specific documents, other than the signed consent, the participant will be referred to by the study participant number/code, not by name. Study documentation will be archived for a period of 5 years according to PC-CTU SOPs.

Source data will be verified as appropriate by the PC-CTU Quality Manager or delegate using a risk-based approach and will be defined in the monitoring plan.

### Data management

All data management functions will be performed in line with PC-CTU SOPs. A Data Management Plan is in place for all PC-CTU studies outlining in detail the study specific procedures that are in place to ensure that high quality data are produced for statistical analysis. The Data Management Plan is reviewed and signed by all applicable parties including the trial manager and the trial statistician prior to the first patient being enrolled.

### Statistical methods

The primary outcome will be analysed using Cox proportional-hazards method, adjusting for practices. Results will be presented as hazard ratios with 95% confidence intervals and associated two-sided *P* values. To test the robustness of the result, a sensitivity analysis will be carried out, using the same method, adjusting the following pre-specified baseline prognostic factors: diastolic and/or systolic BP above or below current NICE target, type II diabetes, and coronary artery disease.

The same approach will be repeated for individual components of the primary composite endpoint and all-cause mortality as secondary analyses. Analyses for other outcomes will be carried out using multiple log-binomial regression models for binary data and linear mixed effect model for continuous data collected over time.

Assumption of proportional hazards will be examined and if any of the assumptions were violated, a suitable alternative survival method will be considered. Similarly, alternative methods will be considered if any violation of assumptions is detected in any of the aforementioned methods for other outcomes.

AEs will be tabulated according to randomised group assignments and the proportions will be compared using Fisher’s exact test.

The primary analyses will be conducted on all randomised participants, applying the principle of intention-to-treat, as far as is practically possible, given any missing data. Specifically, the participants will be analysed in the groups to which they were allocated.

The missing at random assumption will be tested as far as is possible by analysing each baseline covariate in a regression model to determine which, if any, are associated with missingness. All baseline covariates are expected to be observed. Baseline values will be summarised for those who did and did not complete follow-up measurements to describe any characteristics related to missingness that are able to be observed.

We will be analysing our data using an intention-to-treat analysis. All randomised patients will be included in the analysis, assuming non-informative censoring for those withdrawn from the study or lost to follow-up for the primary analysis.

During statistical data review and analysis, any anomalies in the data will be investigated and discussed with the trial management team. The data investigation will be broad and flexible and focus on variability of the data, consistency, dispersion, outliers, inliers, relationships between variables, and relationships over time. The statistical data review will be fully documented with all the output dated. If fraud is proved, fraudulent data will be removed from the analysis.

A full detailed analysis plan, including approach of handling missing data, subgroup analyses, and sensitivity analyses, and a plan for interim analysis will be prepared before the first interim analysis by a statistician who is independent from the study. All analyses will be performed by the trial statistician and validated by a separate statistician. A senior statistician will provide supervision to all statistical aspects in the trial.

### Health economics analysis

The economic evaluation will compare the implementation of ARA plus routine care with routine care for CKD patients. We plan to conduct a within-trial economic analysis. A within-trial cost-consequence analysis will initially be reported, describing all the important results relating to the health care resource use, costs, and consequences of ARA plus routine care compared with routine care for CKD patients. Subsequently, a within-trial cost-effectiveness analysis will consider cost per additional primary endpoint (mortality and onset of CVD) averted, and a cost-utility analysis will determine cost per quality-adjusted life year (QALY) gained.

If trial results demonstrate clinical effectiveness, extrapolation beyond the trial period of 36 months will be undertaken. The methods used will depend on the within trial data, but will either use parametric methods as set out by the NICE Decision Support Unit [52] or use a lifetime decision-model (developing a Markov model or adapting a CKD model), where available, to determine the long-term cost-effectiveness of the intervention in terms of cost per QALY gained. For both the within trial analysis and the model, extensive deterministic sensitivity analysis will be undertaken to assess the impact of changing the key data and will be used to explore the importance of modelling assumptions. Probabilistic sensitivity analyses will be conducted to deal with uncertainty in model parameters and cost-acceptability curves presented.

### Data monitoring

BARACK D will have a DMEC, who will report to and advise the TSC who, in turn, will report to and advise the trial management group. Both the DMEC and TSC will have independent chairs and “stop rule” authority to advise early termination of the trial in the event of safety concerns or futility due to poor recruitment, lack of events, or lack of any treatment effect (“stop rules” to be defined by the DMEC).

### Interim analysis

An internal pilot will be conducted which, in addition to testing study procedures and documentation, will test our assumptions regarding:

i. Practice uptake of the invitation to participate;

ii. Rates of eligible CKD patients in practice populations on existing disease registers;

iii. The response rates to patient invitations;

iv. The rates of consent at baseline visits.

These early recruitment data will be used after 4 months to determine whether any changes are needed to overall recruitment strategy in the other centres, e.g., whether numbers of practice sites need to be supplemented.

A formal futility analysis will be performed at 12 months from first study recruitment with possible termination for safety or futility. “Stop rules” will be defined fully by the DMEC.

### Harms

#### Procedures for recording AEs

All site staff will be appropriately trained in the procedures to follow and the forms to use by the PC-CTU prior to study initiation. Regular central monitoring for all studies and site monitoring, as determined by the trial-specific risk assessment, will be used to ensure that all AEs are identified and acted on appropriately.

All AEs will be recorded at trial visits for the initial 6 months of follow-up by the member of the research team conducting that visit for the previous inter-visit period. Following this initial 6 month period, only the following AEs will be monitored by the member of the research team performing that visit in accordance with PC-CTU SOPs:

• Enlargement of breasts in men and women

• Erectile dysfunction

• Irregular periods

• Vaginal bleeding after the menopause

• Deepening of the voice in women, change in the tone of voice in men

• Excessive hair growth

• Tiredness

• Palpitations

• Numbness and tingling

AEs considered related to the study medication as judged by a medically qualified member of the research team or the sponsor will be followed until resolution or the event is considered stable, clinically insignificant, or asymptomatic. All related AEs that result in a participant’s withdrawal from the study or are present at the end of the study, should be followed-up until a satisfactory resolution occurs.

It will be left to the recruiting clinician’s clinical judgment whether or not an AE is of sufficient severity to require the participant’s removal from treatment and, if treatment is withdrawn, the reason will be recorded. A participant may also voluntarily withdraw from treatment due to what he or she perceives as an intolerable AE. If either of these occurs, the participant must undergo an end of study assessment and be given appropriate care under medical supervision until symptoms cease or the condition becomes stable.

• The severity of events will be assessed on the following scale: 1 = mild, 2 = moderate, 3 = severe.

• The relationship of AEs to the study medication will be assessed by a medically qualified member of the research team.

### Auditing

The study will be conducted in accordance with the current approved protocol, International Conference on Harmonisation Good Clinical Practice (ICH GCP), relevant regulations, and PC-CTU SOPs. The PC-CTU has in place procedures for assessing risk management for trials which will outline the monitoring required. The monitoring will be carried out by the PC-CTU Quality Assurance Manager or equivalent. The investigators and all trial related site staff will receive appropriate training in GCP and trial procedures.

Regular monitoring will be performed according to ICH GCP using a risk-based approach. Data will be evaluated for compliance with the protocol and accuracy in relation to source documents where possible. Following written SOPs, the monitors will verify that the clinical trial is conducted and data are generated, documented, and reported in compliance with the protocol, GCP, and the applicable regulatory requirements. The Study Monitor may also assess serious AEs.

### Ethics and dissemination

#### Declaration of Helsinki

The research team will ensure that this study is conducted in accordance with the principles of the Declaration of Helsinki 1964 (and subsequent revisions).

### ICH guidelines for GCP

The research team will ensure that this study is conducted in full conformity with relevant regulations and with the ICH Harmonised Tripartite Guidelines for Good Clinical Practice (CPMP/ICH/135/95) July 1996.

### Approvals

The protocol, informed consent form, participant information sheet, any further patient facing documents and any proposed advertising material have been submitted to an appropriate Research Ethics Committee (REC - 13/SC/0114), the Medicines and Healthcare Regulatory Authority (MHRA in the UK), the relevant NHS Research and Development Departments, and host institution for written approval. The research team will submit and, where necessary, obtain approval from the above parties for all substantial amendments to the original approved documents.

### Protocol amendments

Any modifications to the protocol which may impact on the conduct of the study, potential benefit of the patient or may affect patient safety, including changes of study objectives, study design, patient population, sample sizes, study procedures, or significant administrative aspects will require a formal amendment to the protocol. Such amendment will be agreed upon by the sponsor, and approved by the Ethics Committee prior to implementation and notified to the health authorities in accordance with local regulations.

Administrative changes of the protocol are minor corrections and/or clarifications that have no effect on the way the study is to be conducted. These administrative changes will be agreed upon by the sponsor, and will be documented in a memorandum. The Ethics Committee may be notified of administrative changes. Previous amendments and protocol versioning are available in online Additional file 1.

### Consent or assent

Informed consent will be taken according to PC-CTU SOPs. A Patient Information Leaflet will be given by the research team to the patient following identification as a potential participant. This leaflet describes the purpose of the study, explains in detail what is required of participants, discusses potential risks and benefits, and provides contact details for the research team. The patient will be given adequate time to consider participation and read the leaflet, consulting with family or friends or any other independent advisors if needed, before seeing the research team for the first study consultation. At the baseline assessment informed consent will be taken, by a suitably qualified member of the research team, who will have received training in GCP and will be authorised to take consent by the Chief Investigator, delegated through the Principal Investigators where applicable. The Consent Form will be signed and dated both by the patient and the member of the research team taking consent. No study related procedures will take place prior to the signing of the consent form. It is clearly stated that the participant is free to withdraw from the study at any time for any reason without prejudice to future care, and with no obligation to give a reason for withdrawal. If the patient requires more time to make a decision on participation then a further consultation will be arranged. Participants will be asked to consent to being contacted by the research team in the event they fail to return for any of the trial follow-up. Consented participants will be asked to complete a Contact Details Form which includes all of their relevant contact details and indication as to their preferred method of contact by the research team. A copy of the signed consent form will be given to the participant and a further copy will be sent with the Contact Details Form to the research team. One copy of the consent will remain in the patient’s records at the recruitment site.

Consent will be taken to allow relevant sections of patient medical notes and data collected during the study to be looked at by responsible individuals from the participating centres, regulatory authorities (including the MHRA), and the NHS Trust, where it is relevant to taking part in the trial.

Patients declining to participate will be asked if they are willing to provide separate written consent to review their records for comparative data. Data will be manually recorded in a separate CRF and transferred to the trial database.

### Ancillary studies

Where transport and local coordination allows an additional blood and urine sample will be taken and stored for future genetic and protein testing.

### Confidentiality

Ensuring patient confidentially is an established and robust process within the PC-CTU. All staff adhere to the principles of GCP and the Data Protection Act, 1998.

It is the PC-CTU’s preferred procedure that patients will only be identified on study documents by use of a unique study ID which cannot be used to identify individual participants. Where this is not possible, specific consent will be taken and participants contact details will be used in order of their preference, e.g., when necessary to make follow-up phone calls or emails. All study documents, such as CRFs, holding patient information are held securely with restricted access either electronically or in paper format.

CRFs and all other documents holding identifiers are anonymised as soon as possible with the process of management being outlined in detail within the ethics application and in trial specific procedures. The holding of patient identifiers is noted, as a trial specific vulnerability in the risk assessment and the Chief Investigator is required to clearly outline how such risks will be managed, to minimise both likelihood and impact and how the success of the management will be monitored and assessed.

### Access to data

The PC-CTU will oversee the intra-study data sharing process, with input from the DMEC. All Principal Investigators will be given access to the cleaned data sets. All data sets will be password protected. To ensure confidentiality, data dispersed will be blinded of any identifying participant information.

### Ancillary and post-trial care

In this population of patients it is thought that if there is no reason to withdraw the ARA from the patient then GPs should keep prescribing it if they wish to.

### Dissemination policy

#### Trial results

The investigators will be involved in reviewing drafts of the manuscripts, abstracts, press releases, and any other publications arising from the study. Authors will acknowledge that the study was funded by the National Institute for Health Research Health Technology Assessment Programme. Reporting will adhere to CONSORT guidelines [53].

#### Authorship

Authorship will be determined in accordance with the International Committee of Medical Journal Editors guidelines and other contributors will be acknowledged.

#### Reproducible research

The WHO dataset is available in online Additional file 1. There are no plans to make the data open source.

### Roles and responsibilities

#### Sponsor

Trial sponsor: University of Oxford

Sponsors Reference: Lock code 107072/414895/1/743

Contact name: Ms Heather House

Address: Research Services, CTRG, Joint Research Office, Block 60, Churchill Hospital, Headington, OX3 7LE

Telephone: +44 (0)1865 572224

Email: heather.house@admin.ox.ac.uk

#### Committees

The PC-CTU Trial Management Committee will be responsible for the monitoring of all aspects of the trial’s conduct and progress and will ensure that the protocol is adhered to and that appropriate action is taken to safeguard participants and the quality of the trial itself. The Trial Management Committee will be comprised of individuals responsible for the trial’s day to day management (e.g., the Chief Investigator, trial manager, statistician, data manager) and will meet regularly throughout the course of the trial.

A TSC will be convened to provide overall supervision of the trial and ensure its conduct is in accordance with the principles of GCP and the relevant regulations. The TSC will review the trial protocol, be notified of any protocol amendments, and provide advice to the investigators on all aspects of the trial. The TSC will consist of members who are independent of the investigators, in particular an independent chairperson.

An independent DMEC will review the accruing trial and safety data to ensure trial site staff and participants are aware of any relevant safety information and to determine whether any reasons exist for the trial to be discontinued.

An independent, blinded Endpoint Committee (blinded to the treatment arm) will adjudicate primary endpoints.

## Discussion

Desirable clinical outcomes for any new therapies would be the effective and safe reduction of cardiovascular events and premature death and/or delay in progression of renal decline. The most important target CKD population for such preventive interventions are those with CKD stage 3b (eGFR 30–44 mL/min/1.73 m^2^), since this has a prevalence at about 1% [1,2], represents progressive renal disease, and is associated with a 12-fold increase in CVD compared to those with eGFR above 60 mL/min/1.73 m^2^[13]. In contrast, relative cardiovascular risk is only 2-fold in patients with CKD stage 3a (eGFR 45–59 mL/min/1.73 m^2^) [13], though the prevalence is near 5% [1,2].

In addition to relative CVD risk reduction, there are limited therapeutic options for the prevention of further renal functional decline in patients with CKD. Presently, the only interventions shown to reduce or prevent renal function decline for most patients with CKD is avoidance of renal damage (e.g., treating infections and avoiding NSAIDs in at-risk people), and effective treatment of risk factors, namely hypertension and diabetes. In addition, drugs acting on the RAAS system offer modest additional benefits to BP lowering alone in patients with diabetic nephropathy with proteinuria [33].

The BARACK D trial evaluates beneficial effects and harms of ARA therapy on CVD risk in patients with stage 3b CKD.

## Trial status

Currently recruiting.

## Abbreviations

ACE: Angiotensin-converting-enzyme; ACR: Albumin/creatinine ratio; AE: Adverse event; ARA: Aldosterone receptor antagonists; ARB: Angiotensin II Receptor Blockers; BARACK D: Benefits of Aldosterone Receptor Antagonism in Chronic Kidney Disease; BNP: B-type natriuretic peptide; BP: Blood pressure; CKD: Chronic kidney disease; CRF: Case report form; CVD: Cardio-vascular disease; DMEC: Data Monitoring and Ethics Committee; eGFR: Estimated glomerular filtration; ESRF: End stage renal failure; GCP: Good Clinical Practice; ICH: International Conference on Harmonisation; LV: Left ventricular; MDRD: Modification of diet in renal disease; MHRA: Medicines and Healthcare Regulatory Authority; NICE: National Institute for Health and Care Excellence; PC-CTU: Primary Care Clinical Trial Unit; PROBE: Prospective randomised open blinded endpoint; QALY: Quality-adjusted life year; SOP: Standard operating procedures; TSC: Trial Steering Committee.

## Competing interests

The authors declare that they have no competing interests.

## Authors’ contributions

NH: design, manuscript writing, critical revision, and final approval of the manuscript. DL: design, manuscript writing, critical revision, and final approval of the manuscript. BT: design, manuscript writing, critical revision, and final approval of the manuscript. RP: data analysis, critical revision, and final approval of the manuscript. JW: data analysis, critical revision, and final approval of the manuscript. PB: data collection, critical revision, and final approval of the manuscript. TB: data collection, critical revision, and final approval of the manuscript. DF: data collection, critical revision, and final approval of the manuscript. PL: data collection, critical revision, and final approval of the manuscript. GF: data collection, critical revision, and final approval of the manuscript. NQ: data collection, critical revision, and final approval of the manuscript. MT: data collection, critical revision, and final approval of the manuscript. JT: conception and design, expert opinion in cardiology, critical revision, and final approval of the manuscript. CF: conception and design, expert opinion in nephrology, critical revision, final approval of the manuscript. RM: conception and design, manuscript writing, critical revision, and final approval of the manuscript. FDRH: conception and design, manuscript writing, critical revision, and final approval of the manuscript. All authors read and approved the final manuscript.

## Supplementary Material

Additional file 1 **REN002 – Appendix – v1.0, doc. Appendix.** WHO Dataset and Protocol versioning. Click here for file
