# Correction: Benefits of Aldosterone Receptor Antagonism in Chronic Kidney Disease (BARACK D) trial–a multi-centre, prospective, randomised, open, blinded end-point, 36-month study of 2,616 patients within primary care with stage 3b chronic kidney disease to compare the efficacy of spironolactone 25 mg once daily in addition to routine care on mortality and cardiovascular outcomes versus routine care alone: study protocol for a randomized controlled trial

**DOI:** 10.1186/s13063-022-06972-9

**Published:** 2022-12-12

**Authors:** Nathan R. Hill, Daniel Lasserson, Ben Thompson, Rafael Perera-Salazar, Jane Wolstenholme, Peter Bower, Thomas Blakeman, David Fitzmaurice, Paul Little, Gene Feder, Nadeem Qureshi, Maarten Taal, Jonathan Townend, Charles Ferro, Richard McManus, F. D. Richard Hobbs

**Affiliations:** 1 Nuffield Department of Primary Care Health Sciences, University of Oxford, Radcliffe Observatory Quarter, Woodstock Road, Oxford, OX2 6GG UK; 2 NIHR Oxford Biomedical Research Centre, Oxford University Hospitals NHS Trust, John Radcliffe Hospital, Headley Way, Oxford, OX3 9DU UK; 3 Department of Public Health, University of Oxford, Old Road Campus, Headington, Oxford, OX3 7LF UK; 4 Centre for Primary Care, Institute of Population Health, University of Manchester, Williamson Building, Oxford Road, Manchester, M13 9PL UK; 5 Primary Care Clinical Sciences, School of Health and Population Sciences, College of Medical and Dental Sciences, University of Birmingham, Edgbaston, Birmingham, B15 2TT UK; 6 Primary Medical Care, University of Southampton, Aldermoor Health Centre, Aldermoor Close, Southampton, SO16 5ST UK; 7 School of Social and Community Medicine, University of Bristol, Office Room 1.01c, Canynge Hall, 39 Whatley Road, Bristol, BS8 2PS UK; 8 School of Medicine, Room 1307 Tower Building, University Park, Nottingham, NG7 2RD UK; 9 Department of Renal Medicine, Royal Derby Hospital, Uttoxeter Road, Derby, Derbyshire DE22 3NE UK; 10 Cardio-Renal Research Group, Departments of Cardiology and Nephrology, Queen Elizabeth Hospital Birmingham and University of Birmingham, Edgbaston, Birmingham, B15 2TH UK

**Correction: Trials 15, 160 (2014)**

**https://doi.org/10.1186/1745-6215-15-160**

Following publication of the original article [1], an error was identified between the primary outcome specified in the original protocol, the detailed SAP, and the endpoint forms and a later version of the protocol that this paper was based upon.

The following paragraph in the “Outcomes” section should be changed as follows:

**Outcomes**

***Primary endpoint***

Time from randomisation until the first occurring of death or hospitalisation for heart disease (coronary heart disease, arrhythmia, atrial fibrillation, sudden death, resuscitated sudden death), stroke, transient ischaemic attack, peripheral arterial disease or heart failure or first onset of any condition listed above not present at baseline. Primary endpoints will be adjudicated by an independent endpoints committee blinded to treatment arm.

Existing Text:

**Outcomes**

***Primary endpoint***

Time from randomisation until the first occurring death, first onset, or hospitalisation for heart disease (coronary heart disease, arrhythmia, new onset/first recorded atrial fibrillation, sudden death, failed sudden death), stroke, or heart failure. Primary endpoints will be adjudicated by an independent Endpoint Committee blinded to the treatment arm.
