# Supplementary material for: Benefits of Aldosterone Receptor Antagonism in Chronic Kidney Disease (BARACK D) trial–a multi-centre, prospective, randomised, open, blinded end-point, 36-month study of 2,616 patients within primary care with stage 3b chronic kidney disease to compare the efficacy of spironolactone 25 mg once daily in addition to routine care on mortality and cardiovascular outcomes versus routine care alone: study protocol for a randomized controlled trial
Source: Trials. 2014 May 8;15:160. doi: 10.1186/1745-6215-15-160 (PMC4113231; doi:10.1186/1745-6215-15-160)
Supplement: Additional file 1 — REN002 – Appendix – v1.0, doc. Appendix. WHO Dataset and Protocol versioning. [file 1745-6215-15-160-S1.doc]

Additional file 1

Appendix A – Administrative information

WHO Trial Registration Data Set

| Primary registry and trial identifying number | EudraCT Number: 2012-002672-13 |
| --- | --- |
| Date of registration in primary registry | 1st June 2012 |
| Secondary identifying numbers | None |
| Source(s) of monetary or material support | National Institute for Health Research – Health Technology Assessment Programme |
| Primary sponsor | University of Oxford |
| Secondary sponsor(s) | None |
| Contact for public queries | BARACK D Trial Team Primary Care Clinical Trials Unit |
|  | Department of Primary Care Health Sciences |
|  | 23–38 Hythe Bridge Street, |
|  | Oxford, OX1 2ET |
|  | barack@phc.ox.ac.uk |
|  | 01865 617884 |
| Contact for scientific queries | BARACK D Trial Team |
|  | Primary Care Clinical Trials Unit |
|  | Department of Primary Care Health Sciences |
|  | 23–38 Hythe Bridge Street, |
|  | Oxford, OX1 2ET |
|  | barack@phc.ox.ac.uk |
|  | 01865 617884 |
| Public title | A potential new treatment for kidney disease |
| Scientific title | Benefits of Aldosterone Receptor Antagonism in Chronic Kidney Disease (BARACK D) Trial – A multi-centre, prospective, randomized, open, blinded end-point, 36-month study of 2,616 patients within primary care with stage 3b chronic kidney disease to compare the efficacy of spironolactone 25 mg once daily in addition to routine care on mortality and cardiovascular outcomes versus routine care alone: study protocol for a randomized controlled trial. |
| Country of recruitment | United Kingdom |
| Health condition(s) or problem(s) studied | Stage 3b chronic kidney disease, mortality, and cardiovascular outcomes |
| Intervention(s) | Active comparator: spironolactone 25 mg once daily in addition to routine care |
|  | Comparator: routine care |
| Key inclusion and exclusion criteria | Inclusion |
|  | • Participant is willing and able to give informed consent |
|  | • Male or female, aged 18 years or above |
|  | • Evidence of stage 3b CKD using the MDRD equation |
|  | • Able and willing to comply with all study requirements, in the recruiting clinician’s opinion |
|  | • Willing to allow his or her General Practitioner and consultant, if appropriate, to be notified of participation in the study |
|  | • Willing to provide contact details to the research team (encompassing recruitment centre and practice staff), for use at any time should the need arise, on trial-related matters |
|  | • If the participant is a female of child-bearing potential, they are willing to ensure effective contraception during the trial period |
|  | Exclusion |
|  | • Female participants who are pregnant, lactating, or planning |
|  | • Pregnancy during the course of the study |
|  | • Type 1 diabetes mellitus |
|  | • Terminal disease or felt otherwise unsuitable by their research clinicians |
|  | • Chronic heart failure clinical diagnosis or known LVSD with ejection fraction <40% |
|  | • Recent myocardial infarction (within 6 months) |
|  | • Alcohol or drug abuse |
|  | • Documented previous hyperkalaemia not thought to be spurious, or intolerance of spironolactone |
|  | • Serum potassium at baseline over 5.0 mmol/L |
|  | • Documented Addisonian crisis and/or on fludrocortisone |
|  | • Documented symptomatic hypotension or baseline systolic blood pressure under 100 mmHg. |
|  | • Recent acute kidney injury or admission for renal failure |
|  | • ACR >70 mg/mmol |
|  | • Prescription of medications with known harmful interactions with spironolactone as documented in the British National Formulary including tacrolimus, lithium, and cyclosporine |
|  | • Any other significant disease or disorder which, in the opinion of the recruiting clinician, may either put the participants at risk because of participation in the study, or may influence the result of the study, or the participant’s ability to participate in the study |
| Study type | Interventional |
|  | Allocation: randomised |
|  | Intervention model: parallel assignment |
|  | Masking: blinded endpoint (outcome assessor) |
|  | Primary purpose: prevention |
|  | Phase III |
| Date of first enrolment | October 2013 |
| Target sample size | 2,616 |
| Recruitment status | Recruiting |
| Primary outcome(s) | Time from randomisation until the first occurring death, first onset or hospitalisation for heart disease (coronary heart disease, arrhythmia, new onset/first recorded atrial fibrillation, sudden death, failed sudden death), stroke, or heart failure. Primary endpoint will be adjudicated by an independent endpoints committee blinded to treatment arm |
| Key secondary outcomes | • Change in blood pressure annually and at final visit |
|  | • Rates of symptomatic hypotension (<100 mmHg systolic or >20 mmHg systolic drop on standing) |
|  | • Changes in BNP |
|  | • Change in ACR |
|  | • Changes in eGFR |
|  | • Change in health related quality of life on EQ-5D-5 L |
|  | • ICECAP-A and QoL VAS |
|  | • Incremental cost effectiveness analysis |
|  | • Transient ischaemic attack – as defined by the American Heart Association (2009) |
|  | • Rates of adverse events |
|  | • Rates of hyperkalaemia |
|  | Intensively phenotyped group |
|  | • Mean change in ambulatory BP from randomisation to final visit (measured in mmHg) |
|  | • Change in carotid-femoral pulse wave velocity from baseline to final visit |

Appendix B – Protocol version

Issue date: 26th November 2013

Protocol Amendment number: 4.0

Author(s): Ben Thompson

Revision Chronology

| Amendment No. | Protocol Version No. | Date issued | Author(s) of changes | Details of changes made |
| --- | --- | --- | --- | --- |
| Ethics meeting response | 2.0 | 21st March 2013 | Ben Thompson | Removal of gift voucher and addition of travel expenses at request of Ethics Committee and addition of “Lay Title”. |
| Substantial amendment number 1 | 3.0 | 7th March 2013 | Ben Thompson | Synopsis – trial phase corrected. |
|  |  |  |  | Synopsis and section 5.2 – primary endpoint clarified. |
|  |  |  |  | Section 5.5 and Schedule – blood sample detail updated. |
|  |  |  |  | Section 5.6 – Home BP measurement section updated following further expert clinical input. |
|  |  |  |  | Section 6.3 – correction of Study Treatment Compliance monitoring detail. |
|  |  |  |  | Section 8 – Statistics section updated and clarified. |
|  |  |  |  | Schedule – Concomitant medications monitoring schedule updated. |
|  |  |  |  | Throughout – minor changes to correct typos and provide clarification. |
| Minor amendment number 1 | 3.1 | 19th September 2013 | Ben Thompson | Change of funder from NIHR School for Primary Care Research to NIHR HTA (confirmed as minor amendment by Ethics Committee co-ordinator). |
| Substantial amendment number 2 | 4.0 | 26th November 2013 | Ben Thompson | Add two questionnaires to measure patient’s overall quality of life. |
